# Highly Efficient and Stable CsPbTh_3_ (Th = I, Br, Cl) Perovskite Solar Cells by Combinational Passivation Strategy

**DOI:** 10.1002/advs.202105103

**Published:** 2022-01-24

**Authors:** Kang Wang, Simin Ma, Xiaoyang Xue, Tong Li, Simiao Sha, Xiaodong Ren, Jingru Zhang, Hui Lu, Jinfu Ma, Shengwei Guo, Yucheng Liu, Jiangshan Feng, Adel Najar, Shengzhong (Frank) Liu

**Affiliations:** ^1^ Key Laboratory of Powder Material & Advanced Ceramics International Scientific & Technological Cooperation Base of Industrial Waste Recycling and Advanced Materials Ningxia Research Center of Silicon Target and Silicon–Carbon Negative Materials Engineering Technology School of Materials Science & Engineering North Minzu University Yinchuan 750021 P. R. China; ^2^ Dalian National Laboratory for Clean Energy iChEM Dalian Institute of Chemical Physics Chinese Academy of Sciences Dalian Liaoning 116023 China; ^3^ Key Laboratory of Applied Surface and Colloid Chemistry Ministry of Education Shaanxi Key Laboratory for Advanced Energy Devices Shaanxi Engineering Lab for Advanced Energy Technology School of Materials Science and Engineering Shaanxi Normal University Xi'an 710119 P. R. China; ^4^ University of the Chinese Academy of Sciences Beijing 100039 P. R. China; ^5^ Department of Physics College of Science United Arab Emirates University Al Ain 15505 United Arab Emirates

**Keywords:** combinational passivation, CsPbTh_3_, efficiency, solar cells, stability

## Abstract

The distorted lead iodide octahedra of all‐inorganic perovskite based on triple halide‐mixed CsPb(I_2.85_Br_0.149_Cl_0.001_) framework have made a tremendous breakthrough in its black phase stability and photovoltaic efficiency. However, their performance still suffers from severe ion migration, trap‐induced nonradiative recombination, and black phase instability due to lower tolerance factor and high total energy. Here, a combinational passivation strategy to suppress ion migration and reduce traps both on the surface and in the bulk of the CsPhTh_3_ perovskite film is developed, resulting in improved power conversion efficiency (PCE) to as high as 19.37%. The involvement of guanidinium (GA) into the CsPhTh_3_ perovskite bulk film and glycocyamine (GCA) passivation on the perovskite surface and grain boundary synergistically enlarge the tolerance factor and suppress the trap state density. In addition, the acetate anion as a nucleating agent significantly improves the thermodynamic stability of GA‐doped CsPbTh_3_ film through the slight distortion of PbI_6_ octahedra. The decreased nonradiative recombination loss translates to a high fill factor of 82.1% and open‐circuit voltage (*V*_OC_) of 1.17 V. Furthermore, bare CsPbTh_3_ perovskite solar cells without any encapsulation retain 80% of its initial PCE value after being stored for one month under ambient conditions.

## Graphical abstract

The guanidinium (GA) cation, acetate (Ac) anion and glycocyamine (GCA) are respectively employed as a dopant, nucleating agent and additive into the CsPbTh3 (Th=I, Br, Cl) perovskite. With this combinational strategy, the CsPbTh3 perovskite solar cell exhibits improved phase stability, lower trap‐assisted recombination and better energy‐level match, resulting in not only higher efficiency of 19.37% but also better ambient stability.

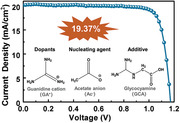

## 1 Introduction

The black phases of CsPbI_3_ have aroused widespread attention in the photovoltaics community due to its exceptional photovoltaic properties such as higher efficiency, excellent chemical and thermal stability, which act as a potential candidate for solar cells.^[^ ^1^, ^2^, ^3^, ^4^, ^5^, ^6^, ^7^ ^]^ In recent years, the power conversion efficiency (PCE) of CsPbI_3_ perovskite solar cells (PSCs) climbed rapidly from 2.9% first reported by Snaith and co‐workers to 20.8% by Liu and co‐workers, and the efficiency gap between the CsPbI_3_ and organic–inorganic hybrid PSCs is further reduced.^[^ ^8^, ^9^, ^10^, ^11^, ^12^ ^]^ Furthermore, CsPbI_3_ has a suitable bandgap (≈1.70 eV) and can be used as the top cell of tandem photovoltaic cells.^[^ ^13^, ^14^ ^]^

Up to date, the introduction of small DMA = (CH_3_)_2_NH_2_^+^ organic cations into precursor solution is essential to approach efficient and stable CsPbI_3_ PSCs.^[^ ^15^, ^16^, ^17^ ^]^ The mechanism was the lattice distortion of *β*‐phase and *γ*‐phase CsPbI_3_ by the decreased bond angle of Pb—I—Pb and increased bond length of Pb—I, resulting in a lower formation temperature of CsPbI_3_ perovskite film.^[^ ^4^, ^18^ ^]^ Several studies have confirmed that DMA cations as the A site additive can induce the formation of intermediate phase during annealing, but they will disappear in the final CsPbI_3_ films due to high temperature and volatility.^[^ ^19^, ^20^, ^21^ ^]^ In other words, it is not the enhancement of tolerance factor that changes the stability of the black phase, but the microstrain that arises from the distortion of corner‐connected [PbI_6/2_]^−^ octahedra in the CsPbI_3_ framework. Knowing that the tolerance factor is a crucial factor in stabilizing the perovskite phase, however, the pristine black CsPbI_3_ phase suffers a low tolerance factor (≈0.807), leading to the thermodynamic stability from the black phase to yellow phase.^[^ ^22^, ^23^ ^]^ Thus, improvement of tolerance factor and regulation of microstrain of CsPbI_3_ perovskite is still an efficient strategy to stabilize the black phase especially for long‐term operation. For instance, Ke et al. stabilized *γ*‐CsPbI_3_ under an appropriate pressure, which revealed that the out‐of‐phase tilt of [PbI_6/2_]^−^ octahedra reduced total energy.^[^ ^24^ ^]^ Steele et al. reported that the introduction of substrate clamping and biaxial strain could induce crystal distortions and texture formation within black CsPbI_3_ thin films, which were thermodynamically stable after being aged under ambient condition.^[^ ^25^ ^]^ Besides, cation engineering such as phenylethylammonium (PEA^+^),^[^ ^26^ ^]^ ethylenediamine (EDA^+^),^[^ ^27^ ^]^ tetrabutylammonium (TBA^+^),^[^ ^28^ ^]^ Ca^2+^,^[^ ^29^ ^]^ Sn^2+[^ ^30^ ^]^ is regarded as an effective way to increase the tolerance factor and reduce the trap state density which can stabilize the black phase and improve the efficiency. We recently found that incorporating a small amount of Br and Cl into CsPbI_3_ film (marked as CsPbTh_3_) exhibited an optimized carrier dynamics performance than pure CsPbI_3_ perovskite due to their higher electronegativity and ionization potential than I.^[^ ^31^ ^]^ Also, Ma et al. reported that introducing Br and Cl into the CsPbI_3_ framework can regulate nucleation, crystallization kinetics, and orientational order within the bulk film. Therefore, the CsPbTh_3_ PSCs achieved a high PCE of 17.14% with an energy loss of 0.6 eV and enhanced phase stability.^[^ ^32^ ^]^ Besides cation engineering, doping and surface passivation using (adamantan‐1‐yl)methanammonium (ADMA), sulfobetaine zwitterion, polymer poly‐vinylpyrrolidone (PVP), and 4‐aminobenzoic acid/teric neostigmine bromide (ABA/NGBr) are also efficient strategies to prevent water molecules in bulk and on the surface of CsPbI_3_ films.^[^ ^33^, ^34^, ^35^, ^36^ ^]^ However, there is a limit to the ability of the single strategy to solve multiple issues in the perovskite film. Therefore, a combinational passivation strategy is needed to improve both efficiency and stability of the PSCs.

In this work, we report a combinational passivation strategy (CPS) to enhance the stability and photovoltaic performance of CsPbTh_3_ inorganic perovskites. Guanidinium cation (GA^+^) was first doped in the CsPbTh_3_ framework to enlarge the tolerance factor to improve its black phase stability. Then, the acetate (Ac) anion was employed as a nucleating agent into GA‐doped CsPbTh_3_ precursor solution to further enhance the thermodynamic stability by creating a slightly distorted PbI_6_ octahedra and suppress the iodine ion migration. Finally, glycocyamine (GCA) was used as a surface termination agent to modify grain boundaries and passivate defects (undercoordinated Pb^2+^ and Cs^+^ vacancy) in the interface. By the coincorporation of GA cation, Ac anion, and GCA into CsPbTh_3_, the perovskite film exhibits higher phase stability and lower trap‐assisted recombination than the control film, as well as better matching of energy levels at the interface between perovskite and charge transport layers. Based on these merits of CPS, the best‐performing CsPbTh_3_ PSC exhibits an efficiency of 19.37% with excellent stability under ambient condition.

## 2 Results and Discussion

**Figure 1:**
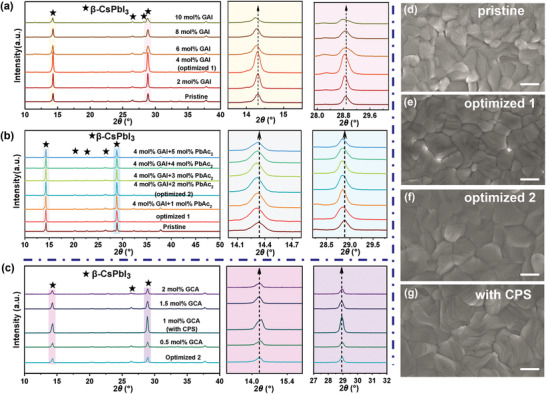
XRD patterns of a) CsGA*_x_* PbTh_3_ (*x* = 0, 0.02, 0.04, 0.06, 0.08, and 0.10), b) CsGA_0.04_PbTh_3_Ac*_x_* (*x* = 0, 0.01, 0.02, 0.03, 0.04, and 0.05), and c) CsGA_0.04_PbTh_3_Ac_0.02_GCA*_x_* (*x* = 0, 0.005, 0.01, 0.015, and 0.02). Typical SEM images of d) CsPbTh_3_, e) CsGA_0.04_PbTh_3_, f) CsGA_0.04_PbTh_3_Ac_0.02_, and g) CsGA_0.04_PbTh_3_Ac_0.02_GCA_0.01_ films, respectively. All the scale bars are 400 nm.

Based on our recent studies, the CsPbTh_3_ film exhibited higher crystallinity than the pure CsPbI_3_ film and achieved outstanding photovoltaic performance.^[^ ^31^ ^]^ In this case, the CPS was performed on the CsPbTh_3_ perovskite film, which significantly enhanced the quality of perovskite film due to the synergistic passivation effect of GA^+^, Ac^−^, and GCA. We first investigate the effect of GA^+^ concentration on the crystal structure of CsPbTh_3_ films. As shown in **Figure** **1**a, X‐ray diffraction (XRD) patterns of all films are tetragonal *β*‐CsPbI_3_ phase, the diffraction peaks at 14.36° and 28.81° are the characteristic of (110) and (220) crystal planes. Notably, the strongest diffraction intensity of (110) peak is observed when the concentration of GA is 4 mol%. Furthermore, the (110) peak slightly shifts toward smaller angles, suggesting that the GA^+^ with larger ionic radius is partially incorporated into the perovskite lattice.^[^ ^37^, ^38^ ^]^ Thus, the 4 mol% GA‐doped CsPbTh_3_ thin film was marked as CsGA_0.04_PbTh_3_ (optimized 1) sample. On this basis, different concentrations of PbAc_2_ (1mol%, 2mol%, 3mol%, 4mol%, 5mol%) were introduced into optimized 1 sample to further optimize crystal structural and electronic properties of CsPbTh_3_ films. As shown in Figure 1b, the peak shift to higher angles with the Ac anion percentage increases, suggesting that incorporation of Ac anion resulted in lattice contraction of optimized 1 sample. However, compared to the pristine film, the CsPbTh_3_ film with GA cation and Ac anion shows a slight expansion of the crystal unit. The CsPbTh_3_ film exhibits the best crystallization when the concentration of Ac anion is 2% (marked as CsGA_0.04_PbTh_3_Ac_0.02_ (optimized 2)). To further evaluate the effect of GA^+^ and Ac^−^ on the crystal structure of perovskite film, density functional theory (DFT) calculations were performed using the Vienna Ab initio Simulation Package (VASP). The simulation result reveals that the coincorporation of GA^+^ and Ac^−^ entails slight local distortions in the CsPbTh_3_ framework compared to the pristine film, as shown in Figure S1 (Supporting Information). An estimation of the relative energy at zero temperature, Δ*E*, for the pure CsPbTh_3_ and optimized 2 sample, were −4.279 and −4.357 eV, respectively, suggesting that optimized 2 sample exhibits better thermodynamic stability (Table S1, Supporting Information). Knowing that vacancy‐mediated diffusion of the iodide ions is the most common process within CsPbI_3_ framework.^[^ ^4^ ^]^ In addition, we examined the iodine ion migration within the CsGA_0.04_PbI_3_Ac_0.02_ framework by DFT calculation, as shown in Figure S2 (Supporting Information), both path‐1 and path‐2 exhibit higher relative energy than the pristine CsPbI_3_, suggesting that the iodine ion migration was effectively suppressed. Except for the crystallographic defects, the passivation of the surface and grain defects is also essential for improving the performance of PSCs. A larger organic molecule GCA was introduced into the optimized 2 film. As presented in Figure 1c, the intensity of (110) and (220) peaks increases with the increase of GCA concentration to 1mol%, and then the intensity decreases. However, the diffraction peak position was not influenced by the introduction of GCA, suggesting that GCA was not doped into the CsPbTh_3_ framework. We speculate that GCA is located at the grain boundaries and surface of the perovskite film. Thus, we expect that GCA can be highly beneficial for passivating the defects at the surface and grain boundaries of the perovskite film.

The scanning electron microscopy (SEM) was carried out to probe the impact of CPS on the morphology of the CsPbTh_3_ perovskite films. As shown in Figure 1d–g and Figure S3 (Supporting Information), all of films exhibit full surface coverage without pinholes. The pristine CsPbTh_3_ film exhibits inhomogeneous grains with an average size of 318 nm, while 4 mol% GA cation incorporation leads to larger average grains (356 nm). Meanwhile, coincorporation of the GA cation and Ac anion increased the uniformity and grain size (377 nm) and reduced the grain boundaries of the CsPbTh_3_ thin film. However, the largest grain size (436 nm) was observed with CPS, indicating that CPS has a positive effect on of the crystallization dynamics and passivation of defects.

**Figure 2:**
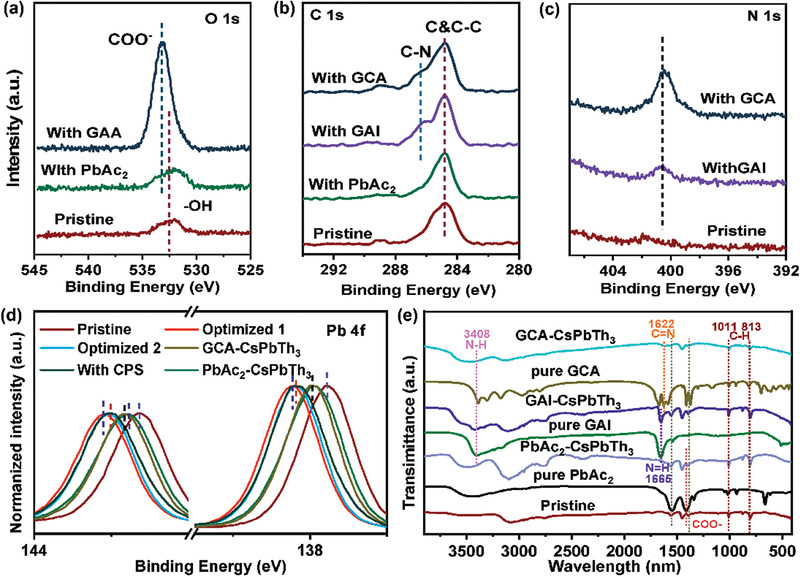
High‐resolution XPS spectra of CsPbTh_3_ with GAI, PbAc_2_, GCA, GAI/PbAc_2_, GAI/PbAc_2_/GCA treatment at the a) O1s, b) C1s, c) N1s, and d) Pb4f regions, respectively. e) FTIR spectra of CsPbTh_3_ treated by GAI, PbAc_2_, GCA, GAI/PbAc_2_, and GAI/PbAc_2_/GCA, respectively.

To probe the role of GA cation, Ac anion, and GCA in the CsPbTh_3_ film, X‐ray photoelectron spectroscopy (XPS) measurements have been performed. **Figure** **2**a shows the O1s spectroscopy of perovskite film with different compounds, only one peak located at 532.6 eV is observed for the pristine perovskite film, which is assigned to the hydroxyl groups or chemisorbed oxygen atoms. The same peak position is detected for the sample with PbAc_2_, combined with the thermogravimetric analysis (TGA) (Figure S4a, Supporting Information), indicating that Ac anion is only involved in the crystallization process and does not exist in the final perovskite film. However, the O1s peak was shifted to 533.2 eV after GCA was introduced into the perovskite film, attributed to the carboxyl group in the GCA molecule.^[^ ^39^ ^]^ Besides, the photoemission spectrum of the C1s of CsPbTh_3_ film (Figure 2b) exhibits a peak position very close to that of pristine perovskite film after introducing the Ac anion into the perovskite precursor solution. Thus, it also reveals that Ac anion does not exist in the final film. Furthermore, the two peaks located at 286.0 eV in C1s and 400.3 eV in N1s (Figure 2c) are attributed to the GA group, combined with the TGA (Figure S4b,c, Supporting Information), indicating that GA cation and GCA additive exist in CsPbTh_3_ film.^[^ ^31^, ^40^, ^41^ ^]^ Notably, as shown in Figure 2d and Figure S5 (Supporting Information), the peak positions of Pb4f, Cs3d, and I3d shift to higher binding energy after coincorporation of GA cation, Ac anion, and GCA, likely due to increased formation energy.^[^ ^39^, ^42^ ^]^ These results are mainly attributed to the GA with six H bonds partially substituting Cs, which can reduce the H—I distance and suppress iodide ion diffusion.^[^ ^40^, ^43^ ^]^ Fourier transform infrared spectroscopy (FTIR) was then used to confirm the above results. As shown in Figure 2e, the pure PbAc_2_ film shows two prominent peaks at 1522 and 1419 cm^−1^. However, the two peaks are absent in CsPbTh_3_ film with PbAc_2_ additive. According to the TGA curve of PbAc_2_, the Ac anion will decompose at 200 °C. However, the annealing temperature of the as‐prepared CsPbTh_3_ films was 210 °C, higher than the decomposition temperature of Ac anion. This result also infers that Ac anion is only involved in the crystallization process and does not exist in the final perovskite film. In addition, the peak around 3408 and 1665 cm^−1^ for pure guanidinium iodine (GAI) powder and GAI‐doped CsPbTh_3_ powder corresponds to N—H bending and N═H stretching vibrations, respectively, indicating that the GA cation is successfully doped into CsPbTh_3_ perovskite lattice.^[^ ^44^, ^45^ ^]^ The peak at 1622 cm^−1^ is related to the C═N stretching vibration of GCA in CsPbTh_3_ film,^[^ ^46^ ^]^ which suppose us to speculate that GCA is at the grain boundaries and on the surface of the CsPbTh_3_ film to passivate the defects.

**Figure 3:**
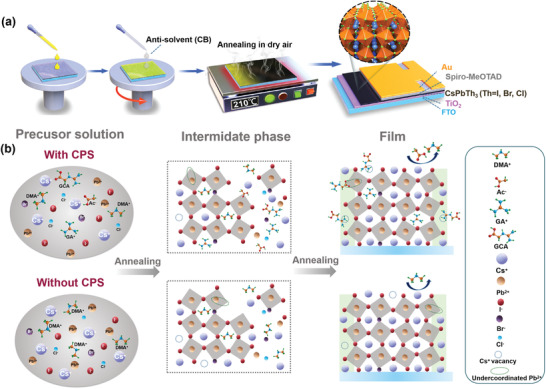
a) Spin‐coating and annealing processes for the control and target CsPbTh_3_ films and schematic of the PSC with the structure FTO/TiO_2_/CsPbTh_3_/spiro‐OMeTAD/Au. b) Schematic diagram of crystal growth with or without CPS.

According to the above analysis, we can determine that the optimal concentration of GAI, PbAc_2_, and GCA are 4 mol%, 2 mol%, and 1mol%, respectively. **Figure** **3**a shows the fabrication process of the CsPbTh_3_ film and the device with a structure of fluorine‐doped tin oxide (FTO)/TiO_2_/CsPbTh_3_/2,2',7,7'‐Tetrakis[N,N‐di(4‐methoxyphenyl)amino]‐9,9'‐spirobifluorene (spiro‐OMeTAD)/Au. We also propose the mechanism of CPS on the phase stability and defect density of CsPbTh_3_ perovskite. For CsPbTh_3_ without CPS (Figure 3b), the low phase stability CsPbTh_3_ film is formed owing to the lower Goldschmidt tolerance factor and more defects such as Cs^+^ vacancy and undercoordinated Pb^2+^ within CsPbTh_3_ film. By incorporation of GA cation, Ac anion, and GCA (with CPS), three proposes have also been achieved. First: the tolerance factor is improved to 0.817 for the CsGA_0.04_PbTh_3_ from its original value of 0.807 for the pristine CsPbI_3_, indicating that the perovskite structure is more stable (Figure S6, Supporting Information). Second: the thermodynamic stability can be improved by slight distortion of [PbI_6_]^4−^ octahedron after incorporating GA cation and Ac anion. Herein, the Ac anion acts as a nucleating agent rather than a dopant, which has been confirmed by the XRD, XPS, FTIR, and TGA characterizations. Third: the undercoordinated Pb^2+^ and Cs^+^ vacancy can be healed by organic cation surface termination (GCA). These three strategies work together to improve the crystalline and stability of perovskite film.^[^ ^43^, ^47^ ^]^ We employed XRD measurement to probe the ambient stability of the CsPbTh_3_ films exposed to ambient with relative humidity (RH) of 20%. As shown in Figure S7 (Supporting Information), the decreased peak intensity of the pristine CsPbTh_3_ film is observed after 10 days of exposure. After introduction of GA cation, Ac anion, and GCA, the peak intensity of the CsPbTh_3_ films does not change considerably upon passivation. We tested the thermal stability of pristine CsPbTh_3_, CsGA_0.04_PbTh_3_, CsGA_0.04_PbTh_3_Ac_0.02_, and CsGA_0.04_PbTh_3_Ac_0.02_GCA_0.01_ films at 100 °C in ambient to further evaluate the phase stability. As shown below in Figure S8 (Supporting Information), the pristine CsPbTh_3_ films quickly turned yellow after 24 h of storage, whereas other three films show much slower degradation, indicating that this path can improve black phase stability. Hence, the enlarged tolerance factor, enhanced thermodynamic stability, and suppressed ion migration are important factors to improve the CsPbTh_3_ phase stability, which is improved by synergistic effect of GA cation, Ac anion, and GCA.

**Figure 4:**
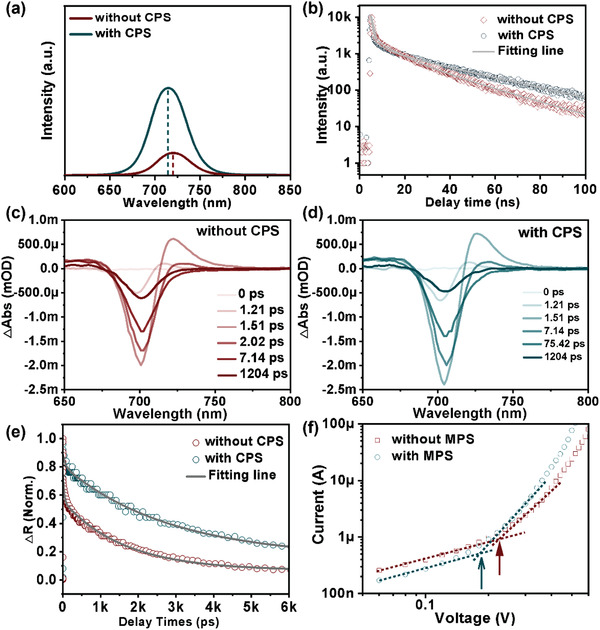
Characterizations of the CsPbTh_3_ with and without CPS. a) PL, b) TRPL curves of the two kinds of films. TA spectra of the CsPbTh_3_ samples c) without CPS and d) with CPS after excitation by a 510 nm laser pulse. e) TA kinetics for two samples. f) Dark space charge limited current (SCLC) curves for electron‐only devices based on the two films.

Furthermore, we carried out the steady‐state photoluminescence (PL) and time‐resolved PL (TRPL) measurements to evaluate the effect of CPS on the optoelectronic properties of the CsPbTh_3_ perovskite film. Note that we prepared the perovskite films on glass substrates with and without CPS. **Figure** **4**a shows a significant increase in optical emission after the CPS treatment. It shows that this CPS can effectively suppress the defect‐induced nonradiative recombination, demonstrating synergistic effect of GA cation, Ac anion, and GCA in CsPbTh_3_ films, as shown in Figure S9 (Supporting Information).^[^ ^48^ ^]^ The optical properties of pristine CsPbTh_3_, GAI–CsPbTh_3_, PbAc_2_–CsPbTh_3_, and GCA–CsPbTh_3_ films are shown in Figure S9 (Supporting Information), the absorbance edge of GCA‐ and Ac^−^‐based CsPbTh_3_ films are similar with the pristine CsPbTh_3_, whereas the GAI–CsPbTh_3_ film exhibits blueshift caused by incorporation of GA cation into the perovskite lattice.^[^ ^49^, ^50^ ^]^ The TRPL decay curves of the CsPbTh_3_ films without and with the CPS were measured in Figure 4b, and the decay times are fitted using equation: *τ*_ave_ = ∑*A_i_τ_i_* ^2^/∑*A_i_τ_i_*.^[^ ^51^ ^]^ As shown in Table S2 (Supporting Information), the average carrier lifetime of the CsPbTh_3_ film is increased from 15.36 to 27.74 ns by the CPS, suggesting that the reduced carrier recombination rate occurred in perovskite film with CPS.^[^ ^52^ ^]^

To further explore ultrafast photoexcited carrier dynamics of CPS‐CsPbTh_3_ film, the characterization of femtosecond transient absorption (TA) spectroscopy was carried out. As shown in Figure 4c,d, at the same delay time under perovskite side illumination, the TA spectra for both the CsPbTh_3_ and CPS‐CsPbTh_3_ films exhibit distinct ground‐state bleaching peak at around 710 nm, indicating 3D film structure. The quenching rate of bleaching peak signal is related to carrier transport efficacy from perovskite to hole transport layer (HTL) or electron transport layer (ETL). The bleaching peaks of CPS‐CsPbTh_3_ exhibited significantly slower decay than the control CsPbTh_3_ film, indicating the reduction of the nonradiative recombination losses within perovskite film, which is consistent with PL and TRPL analyses. These bleach recoveries of CsPbTh_3_ films have been extracted by fitting with double exponential decay kinetics (Figure 4a and Table S3 (Supporting Information)). Without HTL and ETL, the bleach dynamics of CsPbTh_3_ films with and without CPS are attributed to electron–hole recombination rate within perovskite film. The fast and slow time constants for the photobleaching peaks of the CsPbTh_3_ film with CPS are longer than the pristine CsPbTh_3_ film without CPS, indicative of suppressed recombination dynamics of electron–hole pairs within CPS‐CsPbTh_3_ film.^[^ ^31^, ^53^ ^]^

We next quantify the defect in the perovskite layer through space charge limited current (SCLC) method. A typical electron‐only device structure on FTO/TiO_2_/CPS‐CsPbTh_3_/[6,6]‐Phenyl‐C61‐butyric acid methyl ester (PCBM)/Ag was fabricated to measure dark *I*–*V* curves. The trap state density (*n*_trap_) can be calculated by the trap‐filled limit voltage (*V*_TFL_) using the equation: *n*_trap_ = 2*εε*_0_ *V*_TFL_/*eL* ^2^, where the *n*_trap_ is the trap‐state density, *e* is the elementary charge of the electron, *L* is the thickness of perovskite film, *ε* is the relative dielectric constant, and *ε*_0_ is the vacuum permittivity. *V*_TFL_ values of the CsPbTh_3_ film with and without CPS are 0.184 and 0.225 V, as shown in Figure 4f, and, their corresponding *n*_trap_ values were calculated to be 3.10 × 10^15^ and 3.79 × 10^15^ cm^−3^, respectively. These results suggest that the *n*_trap_ was efficiently decreased by the CPS route.

**Figure 5:**
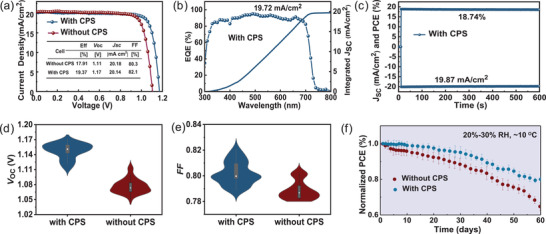
a) *J*–*V* characteristics of PSCs based on the CsPbTh_3_ with and without CPS in the reverse scan. b) EQE spectrum and integrated short‐circuit current density of CPS‐CsPbTh_3_ solar cell. c) Steady‐state output of the champion CPS‐CsPbTh_3_ solar cells. Statistical d) *V*_OC_ and e) FF of PSCs based on CsPbTh_3_ with CPS. f) Long‐term stability of the two devices with and without CPS under ambient conditions (RH: 10–20%).

To evaluate the effect of CPS on the performance of the CsPbTh_3_ PSCs, we fabricated devices with the structure of FTO/TiO_2_/CsPbTh_3_/spiro‐OMeTAD/Au, as shown in Figure 3a. The current density–voltage (*J*–*V*) curves and photovoltaic parameters obtained from the champion cells are shown in **Figure** **5**a and Figure S10 (Supporting Information). The device without CPS exhibits a PCE of 17.91% with short‐circuit current density (*J*_SC_), open‐circuit voltage (*V*_OC_), and fill factor (FF) values of 20.18 mA cm^−2^, 1.11 V, and 80.3%, respectively. After the GA treatment, the CsGA_0.04_PbTh_3_ PSCs render a PCE of 18.32% with a *J*_SC_ of 20.09 mA cm^−2^, *V*_OC_ of 1.15 V, and FF of 79.3%; the PCE of the CsGA_0.04_PbTh_3_ PSCs is further improved to 18.74% with a *J*_SC_ of 20.12 mA cm^−2^, *V*_OC_ of 1.16 V, and FF of 80.3%. Similarly, the best performing device with CPS exhibits a PCE of 19.37% with a *J*_SC_ of 20.14 mA cm^−2^, *V*_OC_ of 1.17 V, and FF of 82.1%. The PCE improvement is mainly attributed to the increase in *V*_OC_ and FF due to the significant reduction of nonradiative recombination of the perovskite film by CPS. The integrated *J*_SC_ from external quantum efficiency (EQE) spectra shown in Figure 5b is 19.72 mA cm^−2^ for the device with CPS, which is well matched with the *J*_SC_ obtained from the *J*–*V* curves.

To confirm the reliability of the *J*–*V* measurements, the champion CPS‐CsPbTh_3_ device delivers a *J*_SC_ of 19.87 mA cm^−2^ and a PCE of 18.74% after 600 s continuous illumination for steady‐state output (Figure 5c). Furthermore, the distributions of photovoltaic parameters (FF and *V*_OC_) to evaluate reproducibility for CsPbTh_3_ devices with and without CPS are shown in Figure 5d,e. Overall, the CPS‐CsPbTh_3_ PSCs exhibit reliability, reproducibility, as well as high solar cell performance. Finally, the device stability was measured under ambient conditions (relative humidity: 10–20%, temperature of ≈20 °C) for over 60 days (Figure 5f). The CPS‐CsPbTh_3_ device retained ≈80% of its initial PCE without encapsulation, while the PCE of CsPbTh_3_‐based PSCs dropped to 65% of its initial PCE. The improved ambient stability indicates that the phase stability and defects/vacancy are suppressed.

**Figure 6:**
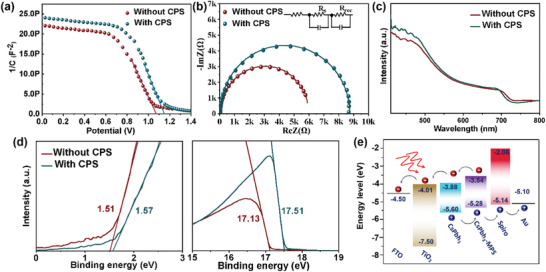
a) Mott–Schottky plots and b) EIS of CsPbTh_3_ cells with and without CPS. c) Absorption spectra and d) UPS spectra of CsPbTh_3_ films with and without CPS. e) Energy diagram of a complete PSC treated by CPS.

To understand the mechanism of improvement of *V*_OC_ and FF by CPS, capacitance–voltage (*C*–*V*) measurements were carried out to further probe the built‐in potential (*V*_bi_) in CPS‐CsPbTh_3_ PSCs and explore the effect of CPS on the separation of photogenerated carriers. As shown in **Figure** **6**a, the *V*_bi_ can be obtained from Mott–Schottky measurement. The *V*_bi_ for the CPS‐CsPbTh_3_ PSC is 1.16 V, higher than the pristine PSC (*V*_bi_ = 1.07 V). The higher *V*_bi_ can be related to the enhanced driving force for the separation of photogenerated carriers, which is favorable for the higher output of the CPS‐CsPbTh_3_ PSCs.

We also probe the interfacial charge transfer kinetics using electrochemical impedance spectroscopy (EIS) measurements. As shown in Figure 6b, the recombination resistance (*R*_rec_) values increased significantly from 5.9 to 8.9 kΩ by the introduction of CPS, suggesting that charge recombination was effectively suppressed. These observations are mainly attributed to suppressing iodine ion migration within the CPS‐CsPbTh_3_ framework and the interfacial passivation (undercoordinated Pb^2+^, Cs^+^ vacancy).

The absorption edges of both CsPbTh_3_ and CPS‐CsPbTh_3_ thin films arise at 721 and 713 nm (≈1.72 vs 1.74 eV), respectively (Figure 6c). Ultraviolet photoelectron spectroscopy (UPS) was also used to measure the energy band levels of CsPbTh_3_ and CPS‐CsPbTh_3_ thin films (Figure 6d). The calculated valance band and conduction band of pristine perovskite film are −5.60 and −3.88 eV, which are upshifted to −5.28 and −3.54 eV after the CPS treatment, as shown in Figure S11 (Supporting Information). The energy gap between the perovskite layer and the spiro‐OMeTAD layer is greatly reduced (Figure 6e), indicating a significant enhancement of the hole transportation at the perovskite/spiro‐OMeTAD interface. Meanwhile, the improved work function increased the driving force for charge separation, leading to an increased *V*_OC_ and FF, which are consistent with the *C*–*V* measurement.

## 3 Conclusion

In summary, we introduced a multifunctional passivation strategy to enhance the CsPbTh_3_ device stability and photovoltaic performance. The GA cation, Ac anion, and GCA were employed as a dopant, nucleating agent, and additive, respectively. The GA^+^ was doped in CsPbTh_3_ to enlarge the tolerance factor and suppress the ion migration, whereas the Ac anion plays a role in enhancing thermodynamic stability by a slight distort of PbI_6_ octahedra of the CsPbTh_3_ framework, which intrinsically improves the black phase stability. Similarly, GCA significantly passivated the surface defects (undercoordinated Pb^2+^ and Cs^+^ vacancy). By the coincorporation of GA cation, Ac anion, and GCA into CsPbTh_3_, the thin film exhibited both phase stability, lower trap‐assisted recombination both in bulk and on the surface, and better matching of energy levels at the interface between perovskite and charge transport layers. With the combinational passivation strategy, the CsPbTh_3_ PSCs exhibited an efficiency of 19.37% with excellent ambient stability. We anticipate that multifunctional management strategy would help increase black phase stability and carrier dynamics for high‐performance perovskite optoelectronics.

## 4 Experimental Section

### Materials

CsI (99.999%), PbBr_2_ (≥98%), PbCl_2_ (≥98%), DMAPbI_3_, spiro‐OMeTAD were purchased from Xi'an Polymer Light Technology Cory. GAI (≥99%) was purchased from Advanced Election Technology Co., Ltd. *N*,*N*‐dimethylformamide (DMF), dimethyl sulfoxide (DMSO), Li‐bis‐(trifluoromethanesulfonyl) imide (Li‐TFSI), and 4‐*tert*‐butylpyridine (TBP, 96%) were purchased from Alfa Aesar. GCA was purchased from Tokyo Chemical Industry Co., Ltd. All chemicals were used as received.

### Device Fabrication

0.6 m triple‐halide perovskite CsPbI_2.85_Br_0.149_Cl_0.001_ (denoted as CsPbTh_3_) was prepared by adding CsI, DMAPbI_3_, PbBr_2_, and PbCl_2_ into mixed solvent (*V*_DMF_/*V*_DMSO_ = 9/1) with molar ratio of 3:2.85:0.149:0.001. First, DMAPbI_3_, PbBr_2_, and PbCl_2_ were dissolved in mixed solvent to ensure good solubility, and then CsI powder was added into the mixed solution. Second, the GA‐doped perovskite solution was prepared by adding a certain amount of GAI (molar ratio with respect to Cs^+^; 2 mol%, 4 mol%, 6mol%, 8mol%, 1mol%) into the precursor solution to form CsGA*_x_* PbTh_3_ films. Third, a certain amount of PbAc_2_ was added into CsGA_0.04_PbTh_3_ precursor to form CsGA_0.04_PbTh_3_Ac*_x_* (*x* = 1 mol%, mol2%, 3 mol%, 4 mol%, 5 mol%) films. Finally, various amount of GCA was added into CsGA_0.04_PbTh_3_Ac_0.02_, the precursor solution. All precursor solutions were stirred at room temperature for 1 h and filtered through 0.2 µm syringe filter. 90 mg of spiro‐OMeTAD, 36 µL TBP, and 22 µL of Li‐TFSI (520 mg mL^−1^) were dissolved in 1 mL chlorobenzene to prepare spiro‐OMeTAD solution.

The FTO glasses were cleaned sequentially with electronic cleaner and water in an ultrasonic bath for 30 min, then treated with UV for 20 min. Then, a 40 nm compact TiO_2_ layer was deposited on the FTO glass by a common sol–gel approach. Then, all the inorganic perovskite precursor solutions were spin‐coated at 1000 rpm for 10 s and 4000 rpm for 30 s. The substrates were annealed at 210 °C for several minutes in ambient environment with low humidity. The spiro‐OMeTAD solution was then spin‐coated on perovskite layer at 5000 rpm for 30 s. Finally, ≈80 nm thick gold top contacts were deposited via thermal evaporation. The device area was confined by a metal mask with an aperture area of 0.09 cm^2^.

### Device Characterization

XRD measurements was carried out at XRD‐6000 with Cu normal focus (NF) radiation (Shimadzu). XPS was performed on a photoelectron spectrometer (ESCALAB Xi+, ThermoFisher Scientific). The surface images were measured by a field emission SEM (ZEISS, SIGMA 500) and the grain size distributions were obtained by Nano measure software. The UV–vis– near‐infrared (NIR) spectra were collected using a UV‐Lambda 2700 spectrometer at 1 nm intervals. Steady PL (excitation at 510 nm) was measured using a FLS980 spectrometer (Edinburgh Instruments Ltd) and TRPL was measured with PicoQuant FluoQuant 300. FTIR spectra were measured with Thermo Scientific Nicolet iS5. TG curves were obtained through TG209F3 from 30 to 800 °C at a rate of 5 °C min^−1^. The *C*–*V* curves were carried out using a Zahner Zennium electrochemical workstation. The band structures of perovskite films were obtained using UPS (ESCALAB 250Xi, Thermo Fisher). The TA spectra were measured by regeneratively amplified Ti:sapphire laser (Light Conversion, 1030 nm, 150 fs, and 100 kHz repetition) and a high‐speed spectrometer (Ultrafast Systems, HELIOS). The monochromator was used by Omni‐*λ*300i series monochromator. The *J*–*V* curves were measured with a solar simulator (SS‐F5‐3A, Enlitech) under AM 1.5G illumination under ambient conditions. The EQE was measured on EnliTech EQE system, including a tungsten‐halogen lamp, a Si detector, and a monochromator. Mott–Schottky curves were obtained on a Zahner MESSSYSTEME PP211 with a step width of 0.01 and a delay of 1 s. The photostability was tested at the maximum power point (1.06 V) for 600 s. The bare solar cells without encapsulation were placed in ambient environment (RH: 15–30%) to test the ambient stability. All calculations were carried out by using the projector augmented wave method in the framework of the DFT, as implemented in the VASP. The generalized gradient approximation and Perdew–Burke–Ernzerhof exchange functional^[^ ^1^ ^]^ were used. The plane‐wave energy cutoff was set to 500 eV, and the Monkhorst–Pack method was employed for the Brillouin zone sampling. The convergence criteria of energy and force calculations were set to 10^−5^ eV per atom and 0.01 eV Å^−1^, respectively. The GA_0.125_Cs_0.875_PbI_2.875_Ac_0.125_ model was built from the 2 × 2 × 2 supercell of CsPbI_3_ by replacing Cs and I with GA and Ac ions, respectively.

## Conflict of Interest

The authors declare no conflict of interest.

## Supporting information

Supporting Information

## Data Availability

The data that support the findings of this study are available from the corresponding author upon reasonable request.

## References

[1] L. Meng , Z. Wei , T. Zuo , P. Gao , Nano Energy 2020, 75, 10486.

[2] K. Wang , Z. Jin , L. Liang , H. Bian , D. Bai , H. Wang , J. Zhang , Q. Wang , S. Liu , Nat. Commun. 2018, 9, 4544. 30382108 10.1038/s41467-018-06915-6 PMC6208436

[3] C. Yi , C. Liu , K. Wen , X. K. Liu , H. Zhang , Y. Yu , N. Fan , F. Ji , C. Kuang , B. Ma , C. Tu , Y. Zhang , C. Xue , R. Li , F. Gao , W. Huang , J. Wang , Nat. Commun. 2020, 11, 4736. 32958808 10.1038/s41467-020-18380-1 PMC7505955

[4] Z. Yao , W. Zhao , S. (F.) Liu , J. Mater. Chem. A 2021, 9, 11124.

[5] Y. Wang , Y. Chen , T. Zhang , X. Wang , Y. Zhao , Adv. Mater. 2020, 32, 2001025. 10.1002/adma.202001025 32964519

[6] M. B. Faheem , B. Khan , C. Feng , M. U. Farooq , F. Raziq , Y. Xiao , Y. Li , ACS Energy Lett. 2019, 5, 290.

[7] X. He , J. Chen , X. Ren , L. Zhang , Y. Liu , J. Feng , J. Fang , K. Zhao , S. F. Liu , Adv. Mater. 2021, 33, 2100770. 10.1002/adma.202100770 34057256

[8] G. E. Eperon , G. M. Paternò , R. J. Sutton , A. Zampetti , A. A. Haghighirad , F. Cacialli , H. J. Snaith , J. Mater. Chem. A 2015, 3, 19688.

[9] B. Yu , J. Shi , S. Tan , Y. Cui , W. Zhao , H. Wu , Y. Luo , D. Li , Q. Meng , Angew. Chem., Int. Ed. 2021, 60, 13436. 10.1002/anie.202102466 33792125

[10] X. Gu , W. Xiang , Q. Tian , S. (F.) Liu , Angew. Chem., Int. Ed. 2021, 60, 23164. 10.1002/anie.202109724 34405503

[11] S. Chen , X. Dai , S. Xu , H. Jiao , L. Zhao , J. Huang , Science 2021, 373, 902. 34413234 10.1126/science.abi6323

[12] C. Liu , C. Igci , Y. Yang , O. A. Syzgantseva , M. A. Syzgantseva , K. Rakstys , H. Kanda , N. Shibayama , B. Ding , X. Zhang , V. Jankauskas , Y. Ding , S. Dai , P. J. Dyson , M. K. Nazeeruddin , Angew. Chem., Int. Ed. 2021, 60, 20489. 10.1002/anie.202107774 PMC8456866 34223674

[13] W. Ahmad , J. Khan , G. Niu , J. Tang , Sol. RRL 2017, 1, 1700048.

[14] K. Xiao , R. Lin , Q. Han , Y. Hou , Z. Qin , H. T. Nguyen , J. Wen , M. Wei , V. Yeddu , M. I. Saidaminov , Y. Gao , X. Luo , Y. Wang , H. Gao , C. Zhang , J. Xu , J. Zhu , E. H. Sargent , H. Tan , Nat. Energy 2020, 5, 870.

[15] Y. Wang , M. I. Dar , L. K. Ono , T. Zhang , M. Kan , Y. Li , L. Zhang , X. Wang , Y. Yang , X. Gao , Y. Qi , M. Grätzel , Y. Zhao , Science 2019, 365, 591. 31395783 10.1126/science.aav8680

[16] Q. Huang , Y. Liu , F. Li , M. Liu , Y. Zhou , Mater. Today 2021, 47, 156.

[17] S. Tan , J. Shi , B. Yu , W. Zhao , Y. Li , Y. Li , H. Wu , Y. Luo , D. Li , Q. Meng , Adv. Funct. Mater. 2021, 31, 2010813.

[18] A. Marronnier , G. Roma , S. Boyer‐Richard , L. Pedesseau , J. M. Jancu , Y. Bonnassieux , C. Katan , C. C. Stoumpos , M. G. Kanatzidis , J. Even , ACS Nano 2018, 12, 3477. 29565559 10.1021/acsnano.8b00267

[19] Y. Wang , X. Liu , T. Zhang , X. Wang , M. Kan , J. Shi , Y. Zhao , Angew. Chem., Int. Ed. 2019, 58, 16691. 10.1002/anie.201910800 31538395

[20] K. Wang , Q. Tian , G. Zhao , J. Wen , J. Huang , C. Gao , Z. Xu , Y. Liu , L. Liang , L. Meng , L. Zhang , Z. Liu , Z. Jin , S. Olthof , S. (F.) Liu , Cell Rep. Phys. Sci. 2020, 1, 100180.

[21] H. Meng , Z. Shao , L. Wang , Z. Li , R. Liu , Y. Fan , G. Cui , S. Pang , ACS Energy Lett. 2019, 5, 263.

[22] M. Saliba , T. Matsui , K. Domanski , J. Y. Seo , A. Ummadisingu , S. M. Zakeeruddin , J. P. Correa‐Baena , W. R. Tress , A. Abate , A. Hagfeldt , M. Gratzel , Science 2016, 354, 206. 27708053 10.1126/science.aah5557

[23] J. Zhang , G. Hodes , Z. Jin , S. F. Liu , Angew. Chem., Int. Ed. 2019, 58, 15596. 10.1002/anie.201901081 30861267

[24] F. Ke , C. Wang , C. Jia , N. R. Wolf , J. Yan , S. Niu , T. P. Devereaux , H. I. Karunadasa , W. L. Mao , Y. Lin , Nat. Commun. 2021, 12, 461. 33469021 10.1038/s41467-020-20745-5 PMC7815753

[25] J. A. Steele , H. Jin , I. Dovgaliuk , R. F. Berger , T. Braeckevelt , H. Yuan , C. Martin , E. Solano , K. Lejaeghere , S. M. J. Rogge , C. Notebaert , W. Vandezande , K. P. F. Janssen , B. Goderis , E. Debroye , Y.‐K. Wang , Y. Dong , D. Ma , M. Saidaminov , H. Tan , Z. Lu , V. Dyadkin , D. Chernyshov , V. V. Speybroeck , E. H. Sargent , J. Hofkens , M. B. J. Roeffaers , Science 2019, 365, 679. 31346140 10.1126/science.aax3878

[26] K. Wang , Z. Li , F. Zhou , H. Wang , H. Bian , H. Zhang , Q. Wang , Z. Jin , L. Ding , S. (F.) Liu , Adv. Energy Mater. 2019, 9, 1902529.

[27] T. Zhang , M. I. Dar , G. Li , F. Xu , N. Guo , M. Grätzel , Y. Zhao , Sci. Adv. 2017, 3, e1700841. 28975149 10.1126/sciadv.1700841 PMC5621977

[28] X. Liu , X. Wang , T. Zhang , Y. Miao , Z. Qin , Y. Chen , Y. Zhao , Angew. Chem., Int. Ed. 2021, 60, 12351. 10.1002/anie.202102538 33760329

[29] H. Zhao , J. Xu , S. Zhou , Z. Li , B. Zhang , X. Xia , X. Liu , S. Dai , J. Yao , Adv. Funct. Mater. 2019, 29, 1808986.

[30] N. Li , Z. Zhu , J. Li , A. K. Y. Jen , L. Wang , Adv. Energy Mater. 2018, 8, 1800525.

[31] K. Wang , C. Gao , Z. Xu , Q. Tian , X. Gu , L. Zhang , S. Zhang , K. Zhao , S. (F.) Liu , Adv. Funct. Mater. 2021, 31, 2101568.

[32] J. Ma , M. Qin , Y. Li , X. Wu , Z. Qin , Y. Wu , G. Fang , X. Lu , Matter 2021, 4, 313.

[33] C. Liu , Y. Yang , O. A. Syzgantseva , Y. Ding , M. A. Syzgantseva , X. Zhang , A. M. Asiri , S. Dai , M. K. Nazeeruddin , Adv. Mater. 2020, 32, 2002632. 10.1002/adma.202002632 32613758

[34] Q. Wang , X. Zheng , Y. Deng , J. Zhao , Z. Chen , J. Huang , Joule 2017, 1, 371.

[35] Y. Wang , G. Chen , D. Ouyang , X. He , C. Li , R. Ma , W. J. Yin , W. C. H. Choy , Adv. Mater. 2020, 32, e2000186. 10.1002/adma.202000186 32363655

[36] B. Li , Y. Zhang , L. Fu , T. Yu , S. Zhou , L. Zhang , L. Yin , Nat. Commun. 2018, 9, 1076. 29540764 10.1038/s41467-018-03169-0 PMC5852044

[37] W. Zhang , J. Xiong , J. Li , W. A. Daoud , Sol. RRL 2020, 4, 2000112.

[38] X. Zhou , L. Zhang , X. Wang , C. Liu , S. Chen , M. Zhang , X. Li , W. Yi , B. Xu , Adv. Mater. 2020, 32, 1908107. 10.1002/adma.201908107 32100401

[39] Z. Zeng , J. Zhang , X. Gan , H. Sun , M. Shang , D. Hou , C. Lu , R. Chen , Y. Zhu , L. Han , Adv. Energy Mater. 2018, 8, 1801050.

[40] A. D. Jodlowski , C. Roldán‐Carmona , G. Grancini , M. Salado , M. Ralaiarisoa , S. Ahmad , N. Koch , L. Camacho , G. de Miguel , M. K. Nazeeruddin , Nat. Energy 2017, 2, 972.

[41] C. Liu , Y. Yang , K. Rakstys , A. Mahata , M. Franckevicius , E. Mosconi , R. Skackauskaite , B. Ding , K. G. Brooks , O. J. Usiobo , J. N. Audinot , H. Kanda , S. Driukas , G. Kavaliauskaite , V. Gulbinas , M. Dessimoz , V. Getautis , F. De Angelis , Y. Ding , S. Dai , P. J. Dyson , M. K. Nazeeruddin , Nat. Commun. 2021, 12, 6394. 34737288 10.1038/s41467-021-26754-2 PMC8568940

[42] J. Liang , Z. Liu , L. Qiu , Z. Hawash , L. Meng , Z. Wu , Y. Jiang , L. K. Ono , Y. Qi , Adv. Energy Mater. 2018, 8, 1800504.

[43] D. W. Ferdani , S. R. Pering , D. Ghosh , P. Kubiak , A. B. Walker , S. E. Lewis , A. L. Johnson , P. J. Baker , M. S. Islam , P. J. Cameron , Energy Environ. Sci. 2019, 12, 2264.

[44] X. Ling , J. Yuan , X. Zhang , Y. Qian , S. M. Zakeeruddin , B. W. Larson , Q. Zhao , J. Shi , J. Yang , K. Ji , Y. Zhang , Y. Wang , C. Zhang , S. Duhm , J. M. Luther , M. Gratzel , W. Ma , Adv. Mater. 2020, 32, 2001906. 10.1002/adma.202001906 32449221

[45] C. Yan , Z. Li , Y. Sun , J. Zhao , X. Huang , J. Yang , Z. Ci , L. Ding , Z. Jin , J. Mater. Chem. A 2020, 8, 10346.

[46] M. Kazemi Miraki , M. Arefi , E. Yazdani , S. Abbasi , M. Karimi , K. Azizi , A. Heydari , ChemistrySelect 2016, 1, 6328.

[47] Q. Yao , Q. Xue , Z. Li , K. Zhang , T. Zhang , N. Li , S. Yang , C. J. Brabec , H. L. Yip , Y. Cao , Adv. Mater. 2020, 32, 2000571. 10.1002/adma.202000571 32449209

[48] M. Mateen , Z. Arain , X. Liu , A. Iqbal , Y. Ren , X. Zhang , C. Liu , Q. Chen , S. Ma , Y. Ding , M. Cai , S. Dai , Sci. China Mater. 2020, 63, 2477.

[49] W. Zhang , J. Xiong , J. Li , W. A. Daoud , J. Mater. Chem. A 2019, 7, 9486.

[50] H. Zhao , Y. Han , Z. Xu , C. Duan , S. Yang , S. Yuan , Z. Yang , Z. Liu , S. (F.) Liu , Adv. Energy Mater. 2019, 9, 1902279.

[51] K. Wang , W. Zhao , J. Liu , J. Niu , Y. Liu , X. Ren , J. Feng , Z. Liu , J. Sun , D. Wang , S. F. Liu , ACS Appl. Mater. Interfaces 2017, 9, 33989. 28914052 10.1021/acsami.7b11329

[52] G. Wu , M. Cai , Y. Cao , Z. Li , Z. Zhang , W. Yang , X. Chen , D. Ren , Y. Mo , M. Yang , X. Liu , S. Dai , J. Energy Chem. 2022, 65, 55.

[53] H. Wang , H. Bian , Z. Jin , H. Zhang , L. Liang , J. Wen , Q. Wang , L. Ding , S. F. Liu , Chem. Mater. 2019, 31, 6231.

