# Supplementary material for: Highly Efficient and Stable CsPbTh3 (Th = I, Br, Cl) Perovskite Solar Cells by Combinational Passivation Strategy
Source: Adv Sci (Weinh). 2022 Jan 24;9(9):2105103. doi: 10.1002/advs.202105103 (PMC8948595; doi:10.1002/advs.202105103)
Supplement: Supplementary file 1 — Supporting Information [file ADVS-9-2105103-s001.pdf]

ADVANCED SCIENCE
Open Access

Supporting Information

for *Adv. Sci.*, DOI: 10.1002/advs.202105103

Highly Efficient and stable $CsPbTh_3$ (Th=I, Br, Cl) Perovskite Solar Cells by Combinational Passivation Strategy

Kang Wang,[1,3] Simin Ma,[1] Xiaoyang Xue,[1] Tong Li,[1] Simiao Sha,[1] Xiaodong Ren,[2*] Jingru Zhang[2], Hui Lu,[1] Jinfu Ma,[1] Shengwei Guo,[1] Yucheng Liu,[2] Jiangshan Feng,[2] Adel Najar,[4] and Shengzhong (Frank) Liu[2,3*]*

Supporting Information

Highly Efficient and stable $CsPbTh_3$ (Th=I, Br, Cl) Perovskite Solar Cells by Combinational Passivation Strategy

Kang Wang,[1,3*] Simin Ma,[1] Xiaoyang Xue,[1] Tong Li,[1] Simiao Sha,[1] Xiaodong Ren,[2*] Jingru Zhang[2], Hui Lu,[1] Jinfu Ma,[1] Shengwei Guo,[1] Yucheng Liu,[2] Jiangshan Feng,[2] Adel Najar,[4] and Shengzhong (Frank) Liu[2,3*]

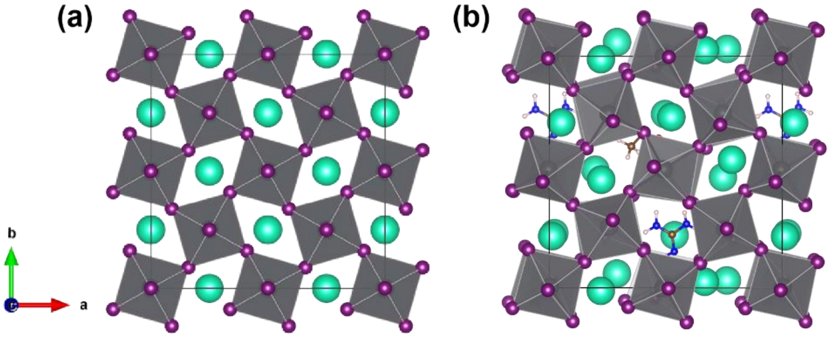

Figure S1. the optimized structures of $CsPbI_3$ and $GA_{0.125}Cs_{0.875}PbI_{2.875}Ac_{0.125}$. The pink, cyan, grey, brown, blue, purple and red balls donate H, Cs, P, C, N, I and O atoms, respectively.

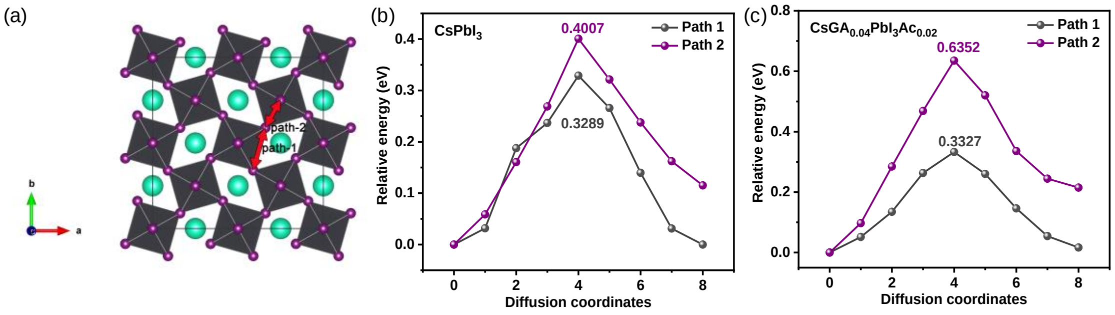

Figure S2. (a) the two paths of iodine ion migration within $CsPbI_3$ and $CsGA_{0.04}PbI_3Ac_{0.02}$ framework, and corresponding relative energy of (b) $CsPbI_3$ and (c) $CsGA_{0.04}PbI_3Ac_{0.02}$.

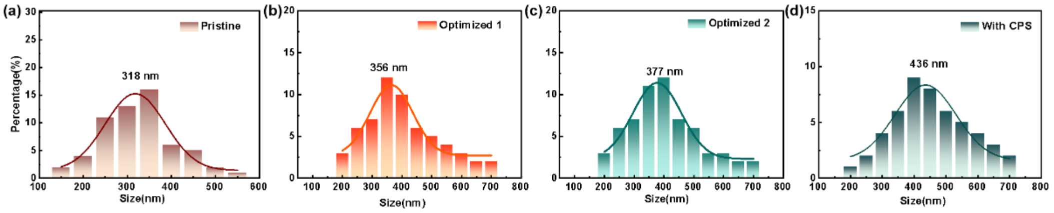

Figure S3. Grain size distributions obtained from (a) $CsPbTh_3$, (b) $CsGA_{0.04}PbTh_3$, (c) $CsGA_{0.04}PbTh_3Ac_{0.02}$ and (d) $CsGA_{0.04}PbTh_3Ac_{0.02}GCA_{0.01}$ films, respectively.

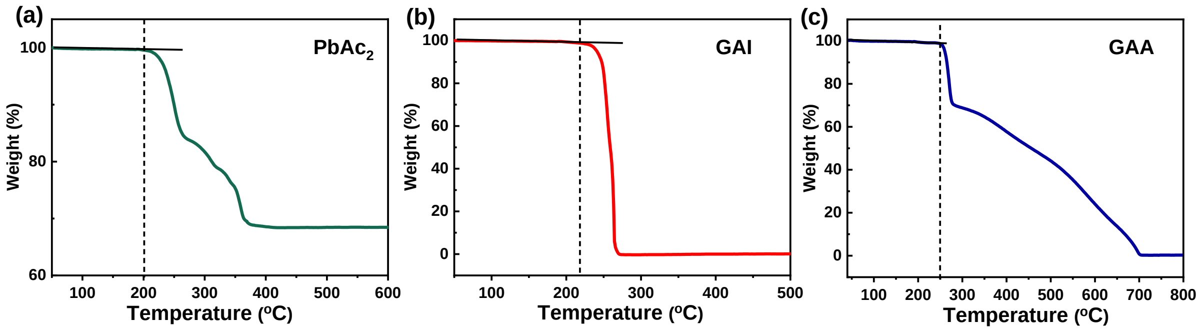

Figure S4. TG analysis of (a) $PbAc_2$, (b) GAI and (c) GCA powders.

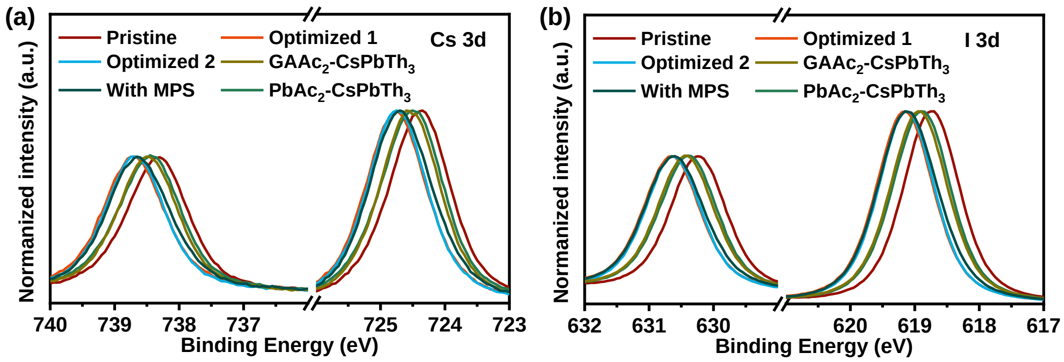

Figure S5. High-resolution XPS spectra of $CsPbTh_3$ with GAI, $PbAc_2$, GCA, GAI/$PbAc_2$, GAI/$PbAc_2$/GCA treatment at the (a) Cs 1s and (b) I 3d region, respectively.

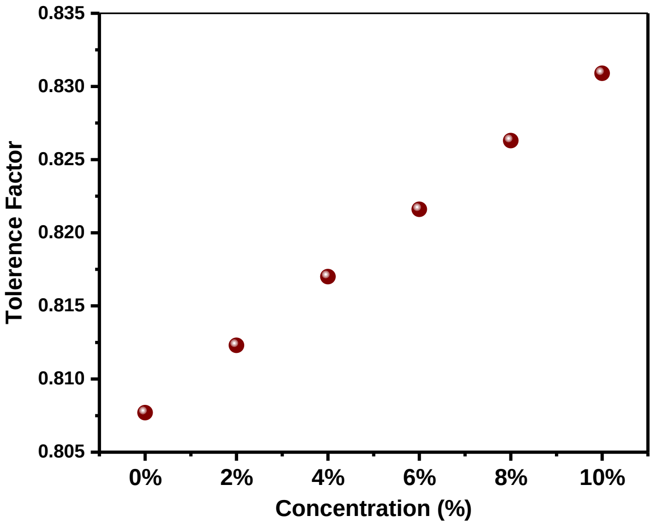

Figure S6. Tolerance factor of $CsPbTh_3$ perovskite with the different concentration GA cation doping.

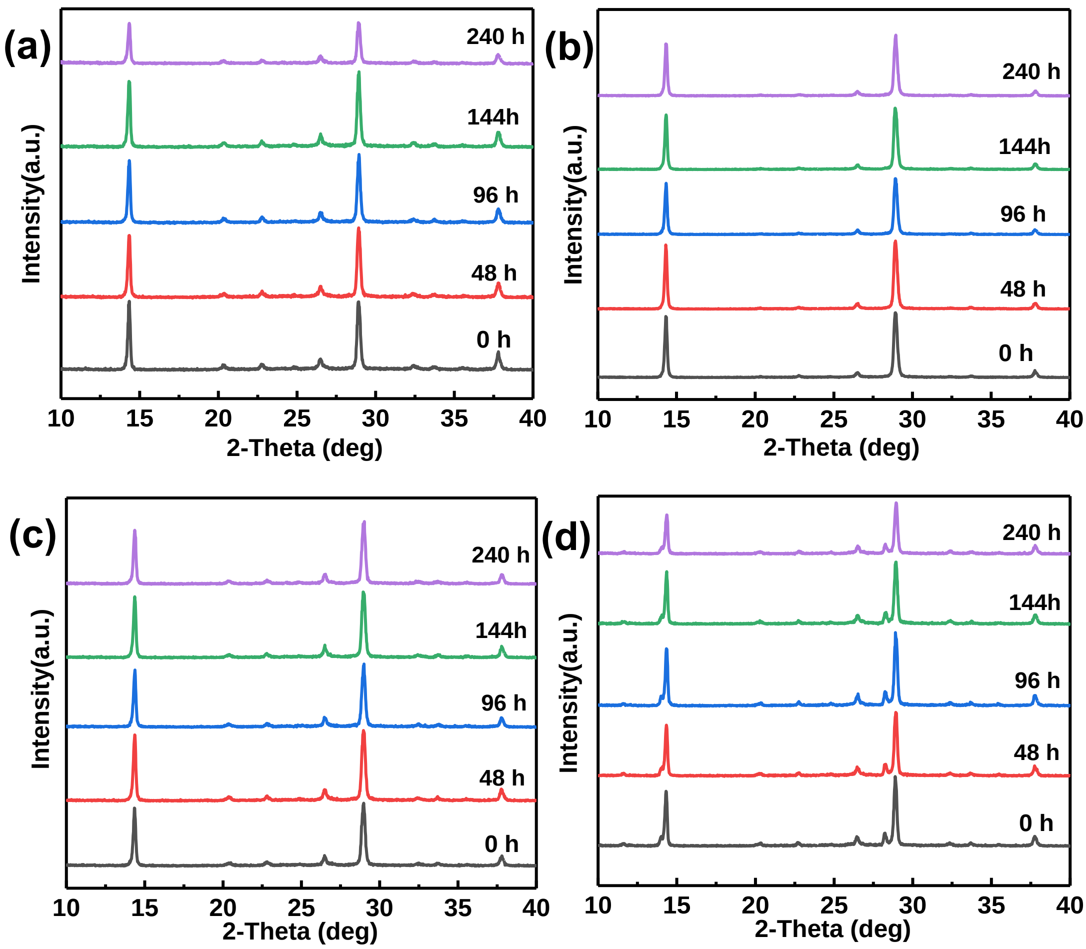

Figure S7. XRD patterns of the (a) $CsPbTh_3$ film, (b) $CsGA_{0.04}PbTh_3$ film, (c) $CsPbTh_3Ac_{0.02}$ film and (d) $CsPbTh_3GCA_{0.01}$ films exposed to a controlled relative humidity (RH) of ~30% in the dark.

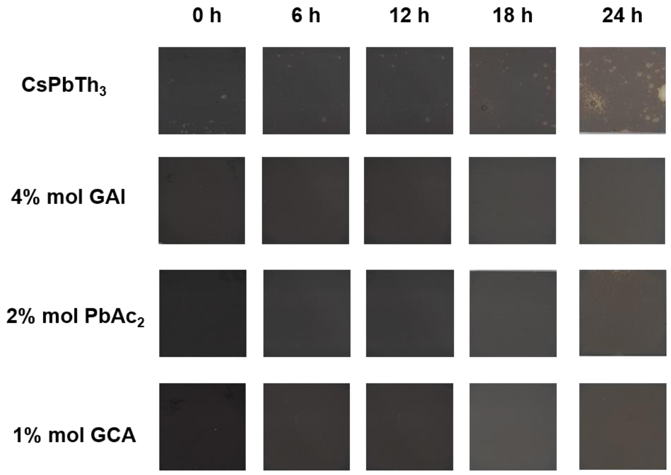

Figure S8. Images of the samples for $CsPbTh_3$ with GAI, $PbAc_2$ and GCA treatment stored at 100 ^{o}C in air.

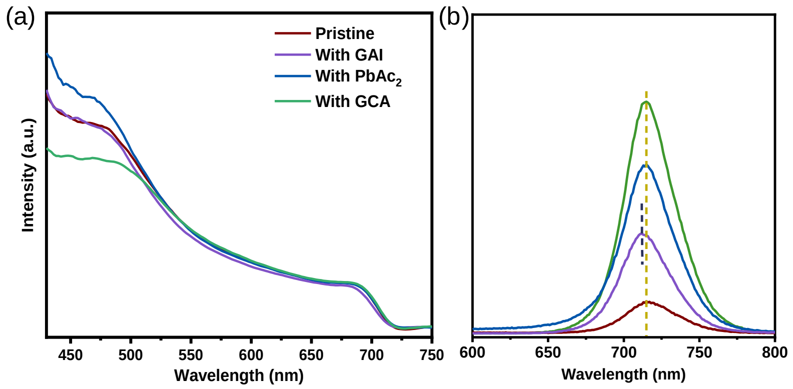

Figure S9. (a) Optical absorption spectra and (b) PL spectra of $CsPbTh_3$ with GAI, $PbAc_2$, GCA treatment, respectively.

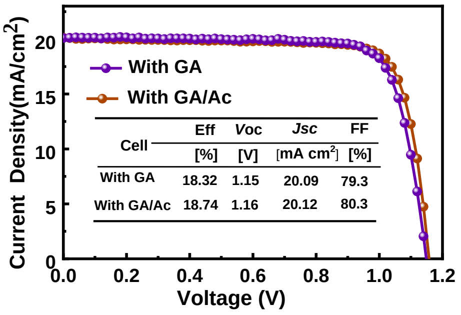

Figure S10. J–V characteristics of PSCs based on the $CsPbTh_3$ with GA and GA/Ac in the reverse scan.

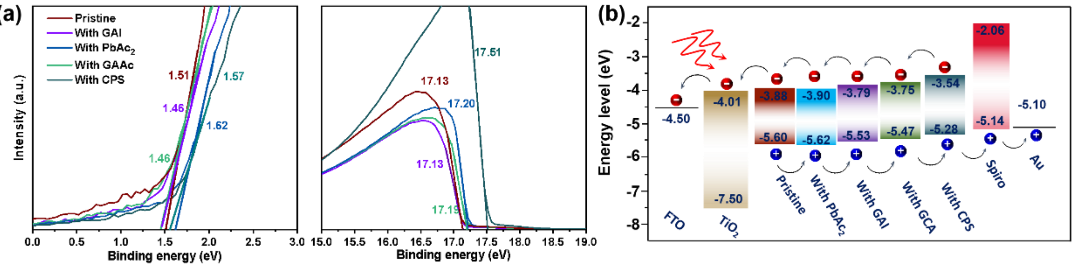

Figure S11. (a) UPS spectra of $CsPbTh_3$ films with GAI, $PbAc_2$, GCA and CPS treatment. (b) Energy diagram of a complete PSC treated by GAI, $PbAc_2$, GCA and CPS, respectively.

Table S1. The relative energy of the $CsPbTh_3$ perovskite films with and without CPS.

**Sample**	**$CsPbI_3$**	**$GA_{0.125}Cs_{0.875}PbI_{2.875}Ac_{0.125}$**
Relative energy (eV/atom)	-4.279	-4.357

Table S2. TRPL parameters of the $CsPbTh_3$ perovskite films with and without CPS.

**Sample**	**τ_{ave} (ns)**	**τ_1 (ns)**	**Amplitude τ_1 (%)**	**τ_2 (ns)**	**Amplitude τ_2 (%)**
$CsPbTh_3$	15.36	1.24	79.53	18.96	20.47
$CsPbTh_3$-CPS	27.74	6.32	49.60	33.54	50.40

Table S3. Fitting parameters for the exciton bleach recovery kinetics of $CsPbTh_3$ films with and without CPS.

**TA-Sample**	**τ_1 (ps)**	**Amplitude τ_1 (%)**	**τ_2 (ps)**	**Amplitude τ_2 (%)**
$CsPbTh_3$-CPS	63.25	10.96	2818.78	89.04
$CsPbTh_3$	33.22	27.94	1614.73	72.06
